# Myosin in chromosome organisation and gene expression

**DOI:** 10.1042/BST20220939

**Published:** 2023-05-12

**Authors:** Isabel W. Shahid-Fuente, Christopher P. Toseland

**Affiliations:** Oncology and Metabolism, The University of Sheffield, Sheffield, U.K.

**Keywords:** gene expression, myosin, nuclear myosin, transcription

## Abstract

The importance of myosin motor protein is well-characterised within the cytoplasm and cytoskeleton. However, mounting evidence on four nuclear myosins highlights the central role these proteins have in maintaining genomic stability and gene expression. This review focuses on each of their critical roles in chromatin structure, chromosome translocation, transcription regulation, and DNA damage repair in terms of maintaining chromosome and chromatin integrity.

## Introduction

**Figure 1.**
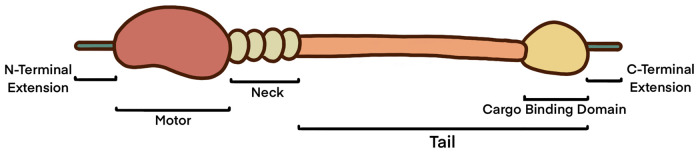
Overview of general nuclear myosin structure. The motor domain is where actin interactions take place. This is commonly where calcium, magnesium, and small molecule binding occurs as well as the autoinhibition of the myosin. The neck domain contains IQ domains which are binding domains for calmodulin and can also be phosphorylated, spliced, and contribute to autoinhibition. The tail domain generally includes a cargo binding domain and a lipid binding domain. Myosin V and VI also have coiled-coil regions, and myosin VI additionally has a single α helix. The tail domain, similar to the motor and neck domain can be phosphorylated and contribute to autoinhibition. Extensions can be found at the N- and C- terminus [3].

Myosins are found throughout eukaryotic cells and are a large superfamily of actin-dependent motor proteins. Conventional myosins are generally responsible for muscle contraction however, it is unconventional myosins which are more abundant with a larger breadth of functions and localisations. Currently, seven myosins have been identified within the nucleus, all roughly following the same conserved structure (Figure 1 and Table 1).

**Table 1:** Nuclear myosins, their isoforms, and their nuclear functions

Myosin	Number of isoforms	Nuclear function
Myosin I	3	**Myosin IB (NMI)**
		Transcription [1,3,10]
		DNA damage repair [1–3,10]
		Chromosome translocation [1,3,10,11]
		**Myosin IA and IC**
		DNA damage repair [3]
Non-muscle Myosin II (NMII)	2	Transcription pre-initiation complex [3,10]
		Embryonic myoblast differentiation [10]
Myosin V	2	**Myosin Va**
		Splicing [10]
		mRNA function [1]
		DNA damage repair [1,2,9,13]
		**Myosin Vb**
		Transcription [1,10]
		DNA damage repair [3]
Myosin VI (MVI)	5	Transcription [3]
		DNA damage repair [3]
Myosin X	1	Mitotic spindle formation [3,10]
Myosin XVI	1	Cell cycle and cell proliferation [3,10]
Myosin XVIII	1	Myofibrillar development [3,10]

Chromosome organisation and gene expression must be tightly regulated to ensure cell survival and genome stability. Dysfunction at any stage can compromise this stability and, in some cases, cause the development of diseases such as cancer [1–7]. Four myosins in particular: myosin I, II, V, and VI have a direct influence on these pathways by regulating DNA damage repair [1–3,8,9], chromatin structure regulation [1,3,10], chromosome movement [1,3,11], and transcription [1–4,12]. This review will compile what is currently known about each of these nuclear myosins, corroborating the importance of myosin in this context.

## Myosin I

The first myosin to be discovered in the nucleus was the myosin I isoform Nuclear Myosin I (NMI). Subsequently, two other isoforms were found: Myosin IA and IC. Being the first, NMI is the most characterised and researched. In both *Drosophila* flies and mammals, NMI is shown to interact with the genome. NMI knockdowns provide evidence of its importance as it leads to the impairment of cell cycle progression, an increase in DNA damage, and an increase in apoptosis [1,14]. The contribution of myosin I on genome integrity is further supported by studies on myosin 1C, which show mutated forms are found in several cancers. Myosin I, mostly when interacting with chromatin remodellers, is established to facilitate chromosomal movement, chromatin rearrangements, transcription and gene expression regulation, and DNA damage repair [1].

### The role of NMI in 3D genome regulation

Genetic information in eukaryotes resides in the nucleus in the form of chromosomes: 3D multi-layered structures of folded genome. The structure is divided into two distinct compartments depending on the type of chromatin. Within these compartments, the chromosome structure is further divided into topologically associated domains (TADs) [1,15].

Each chromosome occupies its own separate chromosomal territory that can be found throughout the nucleus. Gene-poor chromosomes are generally localised around the nuclear envelope and anchored through Lamin B interactions, whereas gene-rich chromosomes reside in a more central position [16]. The territories are not fixed, allowing for long-range chromosome movement, chromatin interactions, and the diffusion of DNA within and between territories [2,3]. NMI facilitates this intracellular movement in response to serum deletion, DNA damage [3], and gene expression [1]. NMI-dependent movement is attributed to actin, Lamin A/C, a filamentous cytoskeletal protein, and Emerin, which bridges that gap between cytoskeleton and nucleus [1,16,17]. However, the exact mechanism and effect of NMI knockout has yet to be established.

Heat shock confirms the interactions of Lamin A/C and NMI by demonstrating their role in the relocation of the Hsp70 gene locus towards the nuclear interior and SC35 nuclear specks to activate Hsp70 gene expression. In turn, recruiting molecular chaperones to protect the DNA [16,17].

Despite NMI function being intact under heat stress, senescence in human dermal fibroblasts (HDFs) shows deregulation in chromosome movement, mirroring results found in serum removal studies. Chromosome 10 is particularly influenced, being found near the nuclear periphery rather than the interior with some genes being down-regulated potentially permanently. One possible explanation could be the alteration of NMIβ localisation. Normally NMIβ can be found throughout the nucleus however, once an HDF cell enters G0 the widespread distribution is lost in favour of large aggregates in the nucleoplasm [11].

TAD organisation is also influenced by NMI. Mouse embryonic stem cell models reveal the binding of NMI to the ISWI chromatin remodeller SNF2H and the Williams syndrome transcription factor (WSTF), forming the complex B-WICH. This complex aids in isolating specific TADs by enabling the recruitment of the insulator protein CTCF [1,10].

### NMI transcription regulation via RNA polymerase interactions

The functions of the B-WICH complex also involve the regulation of the 3D genome, with evidence of a focal role in mRNA and rRNA transcription. It's required for the function of RNA Polymerase I (RNAPI), II (RNAPII), and III (RNAPIII) [1–3].

**Figure 2.**
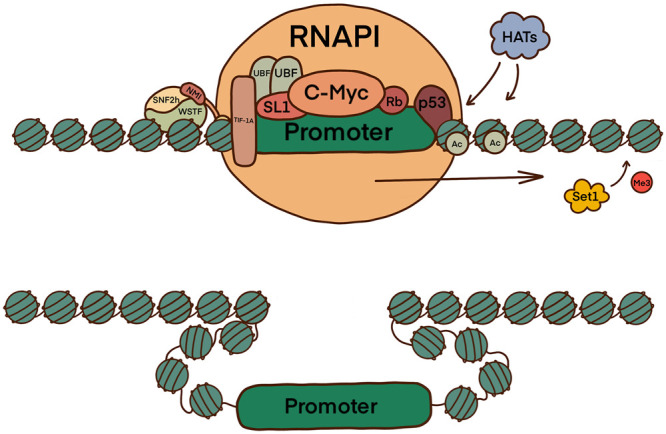
The role of the B-WICH complex in the activation of RNAPI facilitated transcription initiation and elongation. B-WICH connects chromatin to RNAPI and recruits HATs which cause the acetylation of H3 (H3K9Ac) and H4 (H4Ac) histones, rearranging the chromatin to expose the promoter region. This allows for the loading of RNAPI, and interaction with UBF, SL1, TIF-1A, c-MYC, Rb, and p53 to drive transcription.

The interaction of B-WICH with RNAPI activates ribosomal transcription initiation through nucleosome organisation. B-WICH is responsible for the acetylation of H3 and H4, causing the tightly wound chromatin structure to change configuration, exposing a 200 bp region around the rDNA promoter. This occurs due to the recruitment of H3 histone acetyl-transferases (HATs), which directly interact with the NMI tail domain [3]. Histone Methylation Transferase (HMT) Set1/Ash2 are also recruited for the reverse effect [1–2,14]. The exposed promoter region allows for the binding of RNAPI, the transcription factors UBF and SL1, the axillary factor TIF-1A/RRN3, and other transcription regulators (Figure 2) [18,19]. The destabilisation of the B-WICH complex through knockdowns/mutations of the WSTF protein and NMI both greatly reduce transcription. The level of H3K9-Ac declines in WSTF knockdowns, coinciding with the prevention of H3 HATs interacting with rRNA genes, silencing the genes, and reducing transcription. NMI levels were also reduced further contributing to transcription inhibition [20].

**Figure 3.**
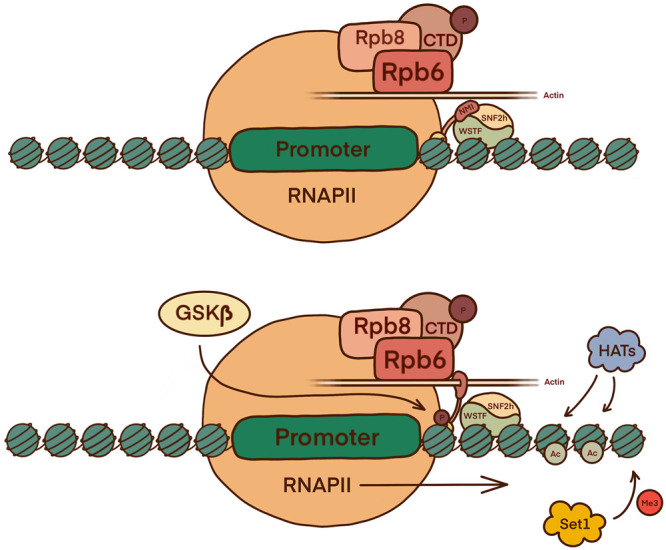
The role of the B-WICH complex in the activation of RNAPII facilitated transcription initiation and elongation. In G1 phase NMI is phosphorylated by GSK3β, resulting in NMI binding to actin in complex with RNAPII. Once bound, NMI associates with the Rpb6 and Rpb8 subunits of the polymerase to load RNAPII onto the chromatin in the correct location. The function of NMI switches when binding with actin-RNAPII is replaced in favour of SNF2h. The B-WICH chromatin remodeller promotes transcription elongation through the same mechanism seen in relation to RNAPI.

NMI is an important component as it forms part of the pre-initiation complex (PIC) and can control RNAPII activation [1,2,21,22]. ChIP-Seq analysis found that NMI binds to the whole mammalian genome with a high correlation to SNF2h, RNAPII, several H3 histone marks, and active class II promoters [21]. Promoters are exposed in the same manner as in RNAPI regulation and phosphorylation on Ser1020 triggers the conformation change necessary for chromatin-bound NMI to load the polymerase onto the promoter. NMI is thought to use a switch mechanism dependent on either actin or SNF2h binding (Figure 3) [1,2,14,21]. There is evidence to suggest that the role of NMI in RNAPII transcription, particularly the recruitment of chromatin-remodellers, is possible through PtdIns(4, 5)P_2_ (PIP_2_) interactions with the NMI C-terminus [23]. Another possible route of PIP_2_ binding is through myosin phosphatase Rho-interacting proteins (MPRIPs) which are a part of the RNAPII/NM1 complex. MPRIPs have been shown to bind to nuclear actin via the N-terminus and can form phase-separated liquid-like condensates via the C-terminus. Both of these functions are speculated to regulate transcription [24,25].

**Figure 4.**
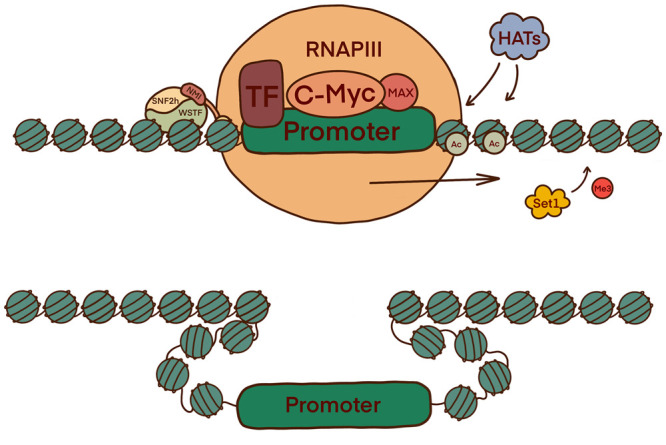
The B-WICH complex in the activation of RNAPIII facilitated transcription initiation and elongation. The promoter regions of 5S rRNA and 7SL RNA are exposed via the same mechanism as shown in Figures 2 and 3. E-boxes (CAGTG) in the IGS region are also revealed, enabling c-Myc-Max to bind and recruit HATs alongside NMI.

Unsurprisingly, the role of NMI in unwinding the chromatin structure at promoter sites for RNAPIII is the same. However, the targets are specifically around the 5S rRNA and 7SL RNA genes. c-Myc-Max subsequently binds and recruits necessary HATs alongside NMI (Figure 4). Additionally, c-Myc can bind to RNAPIII gene promoters through Brf1 in the TFIIIB complex. There is evidence to suggest B-WICH recruited c-Myc during RNAPII transcription also, further interlinking the B-WICH complex role in all the polymerases and emphasising the key role NMI has on transcription initiation and elongation [26].

### Genome integrity: DNA double strand break repair pathway

The genome regulatory functions of the B-WICH complex expand to include DNA double strand break (DSB) repair. Breaks in the DNA can take different forms however, DSBs are the most severe and can form due to various factors including exposure to ionising radiation and chemotherapy drugs [2,27]. The identification of these breaks on actively transcribed template DNA strands triggers a repair pathway which targets the lesions for repositioning and ultimately removal. This mechanism allows cells to progress through the cell cycle with a reduced chance of genomic instability and cancer development [2,27]. Alternatively, if there is extensive DNA damage, cells will undergo apoptosis [28].

**Figure 5.**
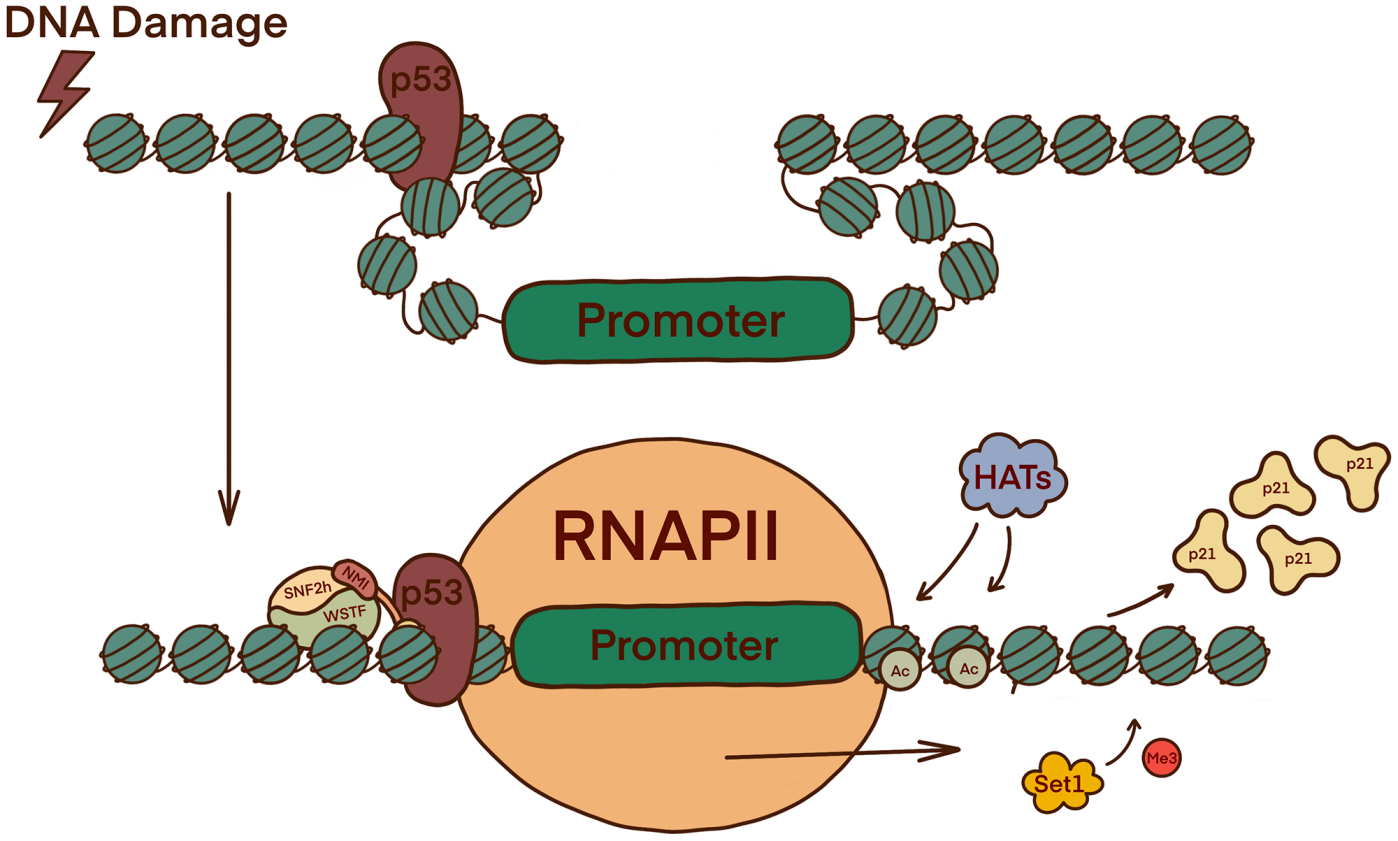
The role of the B-WICH complex in DNA damage dependent cell cycle arrest via the expression of p21. The tumour suppressor p53 is expressed on DSBs are detected and bind to chromatin. For p53 to trigger p21 expression, B-WICH in tandem with RNAPII expose the Cdkn1A gene promoter and bind to the suppressor. Newly synthesised p21 can subsequently trigger G1 cell cycle arrest by (1) inhibiting HATs, and (2) binding to cyclins E and A.

NMI in the B-WICH complex remodels chromatin for the transcription of cell cycle and DNA repair proteins [3,14]. Upon the identification of DSBs, cells must enter cell cycle arrest which can be achieved when the *Cdkn1A* gene is activated to encode the protein p21. The p53 tumour suppressor is activated through a phosphorylation cascade when DSBs are recognised. Although the expression of p53 is not reliant on NMI, the ability of p53 to act as a transcription factor for p21 expression is, and B-WICH with RNAPII is required (Figure 5) [1–3,14]. Knockouts of NMI revealed an absence of cell cycle arrest and cells proliferating at an increased speed with accumulating DNA damage. These effects coincide with the inhibition of p21 expression and an increase in γH2AX, cell cycle proteins, and DNA repair proteins [1,14]. Besides the lack of RNAPII activation, chromatin remodelling, and p21 expression, the absence of NMI would also prevent the long-range movement of chromosomes to allow for the homologous recombination (HR) repair pathway [8,14].

**Figure 6.**
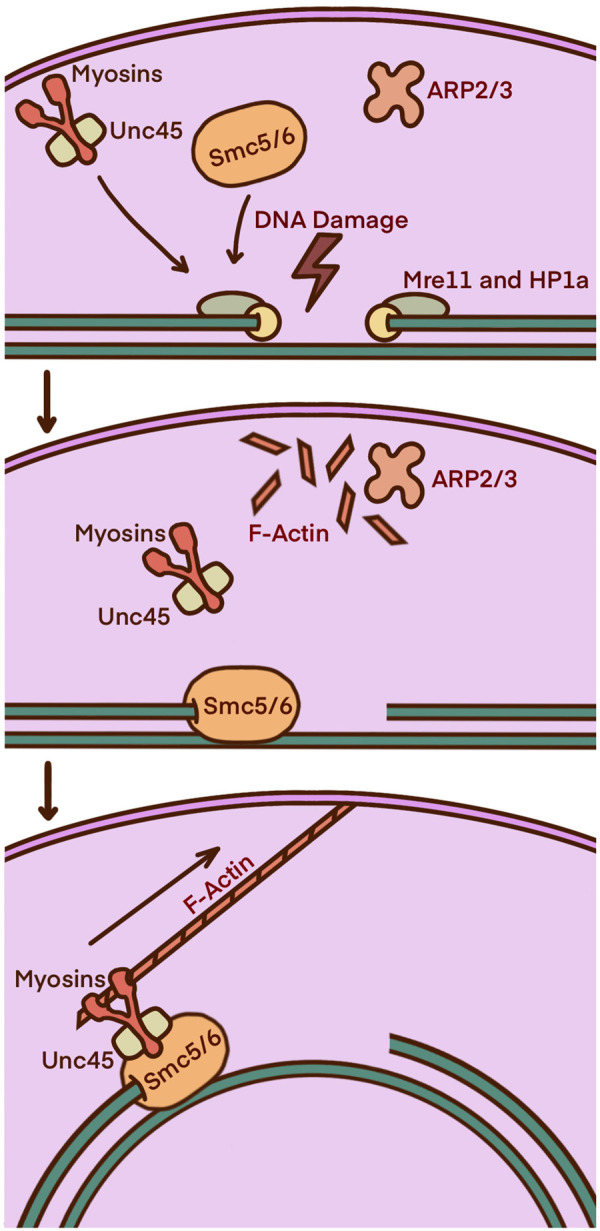
Heterochromatin relocation for HR mediated by NMI, myosin IA, and myosin V. In the S/G2 cell cycle phase, HP1a and Mre11 are attracted to the ends of DSB in heterochromatin, recruiting the ARP2/3 complex and the three relevant myosins. ARP2/3 is responsible for the formation of F-actin filaments which act as a bridge for the break to travel along to get the nuclear periphery. Simultaneously, the myosin activator Unc45 binds to the myosins, recruiting and binding to the Smc5/6 complex which in turn binds to the break site. The myosin motor domains travel along the F-actin filaments taking the breaks to the nuclear pores/membrane proteins for faithful repair.

Around 30% of the human genome consists of ‘silent’ heterochromatin. Due to the high quantity of repeated sequences, DSBs in heterochromatin risk genomic instabilities via chromosome rearrangements and ectopic recombination. To ensure faithful HR, damage sites must first be transported to the nuclear periphery [1,2,8,13,29]. Models using *Drosophila melanogaster* and mouse cells established the relocation mechanism, attributing the ARP2/3 complex, the heterochromatin repair complex Smc5/6, the myosin activator Unc45, and myosins IA, V, and NMI (Figure 6) [1,2,8,9,30,31]. The requirement of myosin was confirmed when depletion led to defective DSB relocation. Mutant studies demonstrate that it is myosin V which is required for movement along the actin filaments. In mouse and fly cells, the absence of myosin triggered IR-induced heterochromatic micronuclei and chromosome abnormalities in the form of fusions, altered number of satellites, and aneuploidies. This particularly affected chromosomes 4 and Y, and the pericentromeric region. Dysfunctional delocalisation was also true for the depletion of Unc45, which is needed for the activation and stabilisation of myosin [8,9].

Two recent paper expands on this model. The first provides more validation into the role of nuclear actin and ARP2/3, along with the activator WASP. They state that nuclear actin filaments allow for chromatin compartmentalisation when DSBs are present, allowing for DSBs to cluster for repair [32]. The other speculates that faithful HR via this model is achieved through phase-separated compartmentalization [24].

Chromosome movement for DSB repair is triggered by specific damage markers, namely γH2AX. H2AX is a histone variant and downstream component of DSB-activated phosphorylation cascades, normally by the phosphatidylinositol 3-kinase-like kinase (PI3KK) family. The phosphorylated form (pSer-139) is known as γH2AX and recruits the PI3KK ATM kinase, resulting in a feedback loop. The accumulation of γH2AX flanking the DSB recruits necessary chromatin remodellers and HR repair proteins, such as BRCA1, RAD51, and RAD52, to the lesion. There is evidence that γH2AX enables NMI-dependent chromosome movements for HR repair. The phosphorylated histone has no noticeable effect on chromatin-associated NMI when DSBs are induced using agents such as cisplatin. However, the inhibition of PI3KKs known to phosphorylate H2AX has an adverse effect on NMI recruitment. Likewise, the same reduction in NMI-chromatin interactions is seen when γH2AX is down-regulated, and both saw hindrance of chromosome movements [3]. Moreover, the importance of NMI in DNA repair was emphasised when knockouts of the myosin caused HR repair to more than half [1,33].

## Myosin VI

Myosin VI (MVI) has multiple isoforms arising from alternative splicing. The resulting variations are referred to by the absence/insertion of amino acid residues in the tail domain. Transcriptional regulation is controlled by the Non-Insert (NI) isoform which can be imported into the nucleus and is associated with cancer development when overexpressed [4]. Both NMI and MVI have overlapping functions in gene expression, but MVI has reverse directionality on actin filaments and reliance on binding factors [3,6,7].

### Transcription regulation with RNAPII

Although not as researched as NMI, the established roles of MVI include transcription initiation and elongation, gene expression and silencing, and chromosome movement. These functions are mostly in conjunction with RNAPII, protein binding partners, and hormone stimulation [3,34].

**Figure 7.**
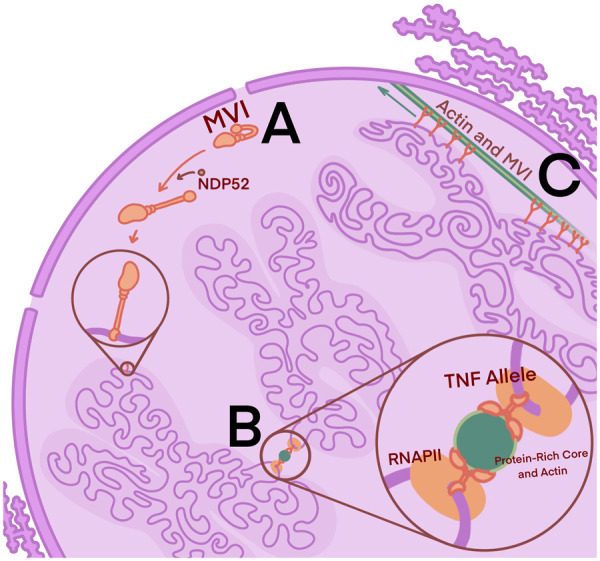
Three functions of MVI in the nucleus. (**a**) Activation of MVI with the binding of NDP52 causing the myosin to unfold. (**b**) TNF alleles in Th1 cells are looped out of their specific territory to a shared area favourable for MVI and RNAPII transcription. (**c**) MVI facilitating long-range chromosome transportation along actin filaments.

Regulation of MVI is controlled by the binding of the transcription co-activator protein NDP52 [4,26] or the tumour suppressor DAB2 [6,35]. In the inactive form, the MVI tail domain is backfolded on itself, preventing chromatin binding. NDP52 binding unfolds the tail, allowing the indirect binding of RNAPII to the motor domain and DNA binding via the C-terminus (Figure 7a) [3,4,6,12].

During initiation, MVI acts as an anchor for transcription factories. These stable clusters of RNAPII and activator proteins reside close to actively transcribed genes and mediate transcription by regulating chromatin interactions [3,12,36]. The use of MVI inhibitor TIP demonstrates a dependence on MVI in the formation of the clusters. Inhibition triggered a significant loss of transcription factories as RNAPII redistributed to the nuclear periphery as well as a reduction in chromatin-bound RNAPII, altogether culminating in a decrease in transcription. For MVI to facilitate RNAPII anchoring, the forces exuded on the motor protein must exceed 2 pico Newtons. Lower forces result in a functional switch to being a molecular transporter [12,34].

The presence of transcription factories allows for RNAPII in a paused state to be loaded to gene promoter sites. Phosphorylation of RNAPII must occur by the Positive Transcription Elongation Factor (P-TEFb) and preliminary findings in T cells suggest that certain genes including *TNF*, *TBX21*, and *TNFAIP8* require MVI for this process [37].

The transcriptional control of MVI for specific genes also includes those regulated by androgen receptors (AR) and oestrogen receptors (ER) [4,35]. For the latter, ER-dependent genes *PS2* and *GREB1* showed a reliance on MVI for gene transcription, with expression dropping by 70–80% and 20–30%, respectively, when ER was impacted using a luciferase assay [4]. ER-positive breast and ovarian cancer cell lines validated these findings. When left untampered, the representative cell line exhibited minimal, if any, DAB2 expression meaning MVI medicated transcription is always active. Reintroduction of DAB2 almost halved *PS2* and *GREB1* expression. This link hints at MVI having an important role in ER signalling and being somewhat regulated by hormone stimulation [3,4,35].

Gene expression mediated by MVI has been linked to two possible binding partners: Heterogenous Nuclear Ribonucleoprotein U (hnRNPU) and nucleolin. Little has been identified in terms of mechanism however, MVI was found to colocalise with both proteins in SC35 nuclear specks and PML nuclear bodies, suggesting a role in pre-mRNA processing and transport, rDNA transcription, rRNA maturation, and chromatin remodelling [3,5].

Investigations into the potential role of MVI in chromosome rearrangements are sparse. One paper discovered knockdowns of MVI cause a significant reduction in the homologous pairing of the *TNF, TNFAIP8,* and *TBET* alleles, as well as RNAPII binding to the gene promoter (Figure 7b) [34,37]. Another provided evidence of MVI-mediated long-range chromatin movement (Figure 7c) [34,38]. MVI movement appears to accelerate when ER-regulated genes were activated. Tracking of chromosomes 2 and 21 found that this increase in motion coincided with the overlap of these chromosomes, each carrying the ER-dependent genes *GREB1* and *TFF1* [38].

## Myosin V

Myosin V has two isoforms (myosin Va and myosin Vb) and is commonly associated with intracellular cargo transportation and exocytosis [2]. Much like MVI, myosin V remains in an inactive folded form, until activated by calcium and binding partners. Adaptor proteins allow for cargo including mRNA, organelles, and vesicles to indirectly bind to the myosin and either be transported short distances with kinesin or long distances with actin filaments [39]. Most nuclear myosins can function as mechanosensory cargo transportation vessels. NMI can carry loads of ∼5 pN [2] whereas both myosin V and VI can carry out regular motor function up to 2pN [2,12,34]. Unlike MVI which switches to an anchoring function when forces exceed 2 pN [12,34], myosin V also experiences halted motion before moving in reverse when forces exceed 3 pN [2]. This transportation role allows myosin V to aid in DNA damage repair alongside NMI by moving heterochromatin breaks (see section Genome Integrity — DNA DSB Repair Pathway) [1,2,8,9,30]. Besides gene relocation, myosin V helps regulate transcription.

### Transcription and gene control of Myosin V

Both isoforms myosin Va and Vb are present in the nucleus of transcriptionally active cells, yet distribution differs slightly. Myosin Va is found throughout in small concentrations excluding the nucleoli, with high concentrations in SC53 nuclear specks, hinting at a possible role in mRNA slicing [40–42]. Transcription inhibition causes myosin Va to relocate to surrounding areas and nucleoli suggesting some form of shuttling mechanism of transcription-related cargo between the two nuclear areas [41]. Lindsay and McCaffrey reported that this isoform does not interact with rRNA nor RNAPI but does with RNAPII [42].

In contrast, myosin Vb localises in the nucleoli, specifically the dense fibrillar component region, where it associates with rDNA, newly synthesised rRNA, β-actin, and RNAPI [1,42]. Minor interactions with RNAPII are also seen. Selective inhibition of RNAPI leads to the redistribution of myosin Vb to nucleoli adjacent ‘plaques' and increased colocalization between polymerase and myosin; something which was not observed when RNAPII was inhibition. This provides evidence to suggest that myosin Vb could be important for RNAPI transcription and movement not dissimilar to the motor function of MVI with RNAPII [42].

## Non-muscle Myosin II

The most researched myosin II is responsible for muscle contractions in skeletal, cardiac, and smooth muscle. However, it is the unconventional nuclear non-muscle myosin II (NMII) that, as the name suggests, resides in the nucleus [43]. Like MVI and myosin V, NMII has autoinhibitory regulation where phosphorylation is needed to unfold and activate the motor protein. In this active state, NMII can aid in regulating transcription and gene expression [43].

### Non-muscle Myosin II mediated gene control

Stem cell commitment requires the activation/silencing of specific genes and is regulated by tissue mechanics. The tissue mechanics necessary take the form of forces exerted on the cell such as compression between cells, and changes to tissue viscosity and stiffness. Mechanical strain has been shown to significantly reduce total mRNA and down-regulate ∼4000 genes. Transcriptional repression was put down to two interconnected factors: the Emerin-dependent compaction and rearrangement of chromatin, and the replacement of global H3K9me2,3 with H3K27me3 [44].

Induced strain in epidermal stem cells coincided with an increase in NMII, F-actin polymerisation, and Emerin at the nuclear envelope, forming a mechanosensory complex [44,45]. Emerin is present throughout the nucleus and links the nuclear lamina to constitutive heterochromatin however, strain causes Emerin in the nuclear interior to move the nuclear periphery reducing Lamin A/C interactions. Centralised Emerin regulates H3K9me2,3, which resides at major satellite regions and Lamin-Associated Domains (LADs). The strain-induced movement coincides with a significant reduction in the histone mark. In turn, the NMII-actin-Emerin complex increases H3K27me3 levels, replacing H3K9me2,3 and mediates gene silencing via the Polycomb Repressive Complex 2 (PRC2) pathway. The histone mark switch also affects chromosome territories of chromosomes 1 and 18, both important for epidermal differentiation, from close to the lamina to a central nuclear position. Transcription is additionally repressed by NMII and Emerin polymerising G-actin outside the nuclear membrane. With G-actin import hindered, RNAPII elongation is hindered, further contributing to the accumulation of H3K27me3 at silenced genes [44].

NMII has also been linked to the assembly of the PIC and the regulation of *ICAM-1* transcription [38,46]. The mechanism is not dissimilar to that of myosins I/VI/V. A study in primary smooth muscle cells discovered that overexpression of NMII causes an increase in ICAM-1 protein expression. NMII could be related to RNAPII binding as immunoprecipitation found associations with RNAPII, α-actin, β-actin, and TFIIB. A model has been put forward where phosphorylation of NMII causes the motor function to distance DNA and RNAPII, reducing transcription. Dephosphorylation has the reverse effect [46].

## Discussion and conclusion

The evidence put forward in this paper, along with the complexity of the transcription and gene expression process, starts to form a comprehensive picture of how all four of these nuclear myosins function in an interconnected system. These myosins have similar compositions, perhaps explaining the overlap and potentially revealing new functions that have yet to be explored.

Although a lot of the mechanisms are yet to be fully established, the four myosins are linked to transcriptional regulation. Some mechanisms are unique to specific myosins such as NMI in B-WICH but both NMI and MVI appear to work simultaneously in transcription initiation and elongation. It would be interesting to verify if one model is preferential to the other. If one is removed, does the other compensate and if so, to what extent? mRNA transport and processing is another aspect that could be investigated as localisation in the SC53 nuclear specks seems to be a common crossover.

Parallels can also be drawn between the components required for long-range chromosome movements which could lead one to speculate that the underlying mechanisms underlying are similar if not the same. Certain myosins might be responsible for the relocation of different chromosomes e.g. NMI for chromosome 1, 10, and 18, and MVI for chromosomes 2 and 21. Continuing this research could highlight which myosins influence which chromosomes, and how they work together.

Chromatin rearrangements to both facilitate transcription and DNA damage repair are among the myosin roles with the least amount of research, with NMI and myosin V having the most established links. So far little overlap between the nuclear myosins have been seen in this regard except for both myosin V and NMI in HR repair. Many papers have looked into the transcriptional response of MVI to DNA damage and early findings have hinted at MVI being capable of chromatin rearrangement, therefore there could be a possibility of MVI also moving heterochromatin breaks or relocating chromatin for another purpose. Moreover, MVI localises on PML nuclear bodies further indicating a potential chromatin remodelling role.

Finally, it may be beneficial to analyses the DNA binding sites of the nuclear myosins using techniques such as ChIP-Seq to assess specific gene interactions. Currently, only NMI and MVI have been analysed in such a way, with only NMI being sequences as a whole. This could discover new associations and also provide verification to some studies that have linked some myosins to specific genes e.g. MVI with ER-dependent genes.

Myosins have previously been shown to work together in a wide range of cytoplasmic roles such as endocytosis [47]. Given these prior findings it would be expected for same to be true in the nucleus. Although mechanisms need to be characterised and more research is required there is significant evidence that NMI, NMII, myosin V, and MVI work in tandem to maintain genome integrity and stability.

## Perspectives

- The correct organisation of the genome and regulation of gene expression are critical for cellular function and health. Nuclear myosins underpin this activity therefore their function and regulation need to be defined.
- Nuclear myosins form direct interactions with transcription regulators and RNA Polymerases. They act to support activity of regulators and can physically hold RNA polymerase complexes at promoters. Moreover, nuclear myosins enable movement of chromosomes.
- The molecular detail on the action of nuclear myosin is required to understand their function. Moreover, information needs to be integrated between gene expression, DNA repair, replication and chromatin organisation fields with the cytoskeletal field to identify wider myosin functions within the nucleus.

## Open Access

Open access for this article was enabled by the participation of University of Sheffield in an all-inclusive *Read & Publish* agreement with Portland Press and the Biochemical Society under a transformative agreement with JISC.

## Abbreviations

AR: androgen receptors
DSB: double strand break
HATs: histone acetyl-transferases
HDFs: human dermal fibroblasts
HR: homologous recombination
MPRIPs: myosin phosphatase Rho-interacting proteins
MVI: Myosin VI
NMI: Nuclear Myosin I
NMII: non-muscle myosin II
PI3KK: phosphatidylinositol 3-kinase-like kinase
PIC: pre-initiation complex
PIP_2_: PtdIns(4, 5)P_2_
RNAPI: RNA Polymerase I
RNAPII: RNA Polymerase II
RNAPIII: RNA Polymerase III
TADs: topologically associated domains
WSTF: Williams syndrome transcription factor
